# The Power of Movement: Linking Physical Activity with Nutritional Health and Blood Sugar Balance in a Dalmatian Type 2 Diabetic Population

**DOI:** 10.3390/nu17010187

**Published:** 2025-01-04

**Authors:** Josipa Radić, Andrej Belančić, Hana Đogaš, Marijana Vučković, Tina Đogaš, Leida Tandara, Marina Grubić, Lucija Šolić Šegvić, Ivana Novak, Mislav Radić

**Affiliations:** 1 Department of Internal Medicine, Division of Nephrology, Dialysis and Arterial Hypertension, University Hospital of Split, 21000 Split, Croatia; josiparadic1973@gmail.com (J.R.); mavuckovic@kbsplit.hr (M.V.); tina.dogas@gmail.com (T.Đ.); lucijasolicc@gmail.com (L.Š.Š.); ivana.i.novak@gmail.com (I.N.); 2 Internal Medicine Department, School of Medicine, University of Split, 21000 Split, Croatia; 3 Department of Basic and Clinical Pharmacology and Toxicology, Faculty of Medicine, University of Rijeka, 51000 Rijeka, Croatia; andrej.belancic@uniri.hr; 4 Department of Neurology, University Hospital of Split, 21000 Split, Croatia; hana.dogas@gmail.com; 5 Division of Medical Laboratory Diagnostic, University Hospital of Split, 21000 Split, Croatia; 6 Department of Medical Chemistry and Biochemistry, School of Medicine, University of Split, 21000 Split, Croatia; 7 Institute for Emergency Medicine of Split-Dalmatia County, 21000 Split, Croatia; marina.grubic123@gmail.com; 8 Department of Internal Medicine, Division of Rheumatology, Allergology and Clinical Immunology, University Hospital of Split, 21000 Split, Croatia

**Keywords:** physical activity, body composition, type 2 diabetes mellitus, Mediterranean diet

## Abstract

Background and Objectives: Regular physical activity (PA) and Mediterranean diet (MeDi) adherence independently improve glycemic control and clinical outcomes in type 2 diabetes mellitus (T2DM). This study examined the associations between PA, body composition (BC), MeDi adherence, and glycemic control in Dalmatian T2DM patients. Materials and Methods: A cross-sectional study was conducted at the University Hospital of Split (November–December 2023) during an open call for T2DM patients. Data collected included blood/urine samples, blood pressure, BC, and anthropometrics. MeDi adherence and PA were assessed via the Mediterranean Diet Service Score and the International PA Questionnaire-Short Form. Results: Among 252 participants (median age: 67 years, IQR: 60–73; 51.6% women; median T2DM duration: 10 years, IQR: 6–20), PA levels were low (31.4%, N = 79), moderate (45.2%, N = 114), and high (23.4%, N = 59), with uniformly low MeDi adherence across groups. Low PA was associated with higher body mass index (BMI) and lower phase angle (PhA). PA negatively correlated with fat mass (FM; %) and visceral adiposity. Positive BMI predictors included FM (kg), total body water, visceral fat level, and PhA, while fat-free mass, intracellular water, and FM (%) were negative predictors. The estimated glomerular filtration rate was the only positive predictor of the total metabolic equivalent of the task score. Conclusions: PA enhances BC and metabolic health, but inadequate MeDi adherence limits these benefits in the T2DM population. To optimize glucose control and health outcomes, public health initiatives must emphasize MeDi adherence and a combination of aerobic and resistance training.

## 1. Introduction

Diabetes mellitus is a chronic metabolic disorder caused by defects in insulin secretion and/or insulin action, which results in hyperglycemia; prolonged hyperglycemia can lead to acute complications (e.g., diabetic ketoacidosis), chronic complications (e.g., retinopathy, chronic kidney damage, diabetic foot ulcers), and consequently, impaired quality of life (QOL) and reduced life expectancy. Type 2 diabetes (T2DM) is a chronic global epidemic that affects 537 million adults worldwide and is expected to rise to 783 million by 2045 [1]. Around 50% of cases remain undiagnosed and 6.7 million people die from diabetes each year, many of them under the age of 60 [1]. T2DM accounts for 90% of diabetes cases, affecting both overweight and normal-weight individuals, with the latter group having a higher risk of death due to sarcopenia [2].

A sedentary lifestyle can exacerbate the challenges of diabetes management, leading to suboptimal blood glucose control and an increased risk of diabetes-related complications [3]. Exercise interventions are an important tool in the prevention and management of T2DM and can improve a wide range of cardiovascular and metabolic outcomes [4,5]. The benefits of exercise in preventing and managing diabetes have been demonstrated in numerous studies, particularly in improving glycemic control, enhancing body composition, and preventing or delaying diabetes-related complications [6]. Structured lifestyle intervention trials that include physical activity (PA) of at least 150–175 min/week and dietary energy restriction targeting weight loss of 5–7% have demonstrated reductions of 40–70% in the risk of developing T2DM in individuals with prediabetes [7]. The World Health Organization (WHO) emphasizes PA as one of the fundamental pillars for the treatment of T2DM, along with diet and medication, and, therefore, a minimum of 150 min of moderate-intensity aerobic PA or 75 min of vigorous aerobic PA per week is prescribed for adults [3,4].

Regular exercise promotes adaptations in adipose tissue and liver that further enhance insulin sensitivity [8]. Repeated exercise sessions over a longer period induce favorable changes in body composition comprising muscle and adipose tissue mass changes, intramyocellular lipid deposition, hepatic and blood lipid levels, and whole-body resting and meal-associated insulin signaling [9]. Indirect mechanisms, such as increased energy consumption and improved body composition, reduced adipose tissue inflammation and ectopic lipid deposition in the liver, and enhanced metabolic capacity of the skeletal muscles, may subsequently lead to direct effects on insulin signaling in target tissues [9].

For most patients living with diabetes, physical training also improves muscle mechanics, muscle insulin sensitivity, and glucose utilization through mitochondrial oxidation and storage as muscle glycogen [7,10]. However, the optimal exercise prescription to maintain or improve the health status of the T2DM population remains uncertain [4,5]. Although there is no international consensus on a correct method to describe PA levels derived from questionnaires or self-assessment surveys, the International Physical Activity Questionnaire-Short Form (IPAQ-SF) has already been used in the assessment of diabetic populations [11,12,13].

In addition to PA, the Mediterranean diet (MeDi), one of the best-researched diets, has shown great benefits in improving insulin resistance in obesity [14]. To deduce, MeDi is a traditional dietary pattern rooted in the cultures of the Mediterranean region, characterized by its emphasis on plant-based foods such as vegetables, fruits, grains, nuts, and legumes, moderate consumption of fish, seafood, and dairy, limited intake of meat and alcohol, and a high reliance on olive oil, with specific food choices and preparation methods varying across the diverse countries and cultures of the area, making it both a cultural cornerstone and one of the healthiest dietary patterns globally. Current research shows that the MeDi significantly improves glucoregulation and body composition in overweight/obese individuals [15]. A recent umbrella review of dietary interventions and nutritional factors for the prevention of T2DM also suggests that MeDi and interventions that alter the quality of diet intake significantly reduce the risk of T2DM [16]. According to meta-analytic data, MeDi was associated with better glycemic control in T2DM and prediabetes compared to lower-fat diets, suggesting that it is suitable for T2DM management [17].

Our previously published study of Dalmatian patients living with T2DM showed a significant gap in awareness of kidney disease (KD). Only 6% of participants believed they had KD, although 31% of participants were diagnosed with KD after clinical testing given the high prevalence of obesity and low adherence to the MeDi in this population [18]. Considering all these uncertainties, the aim of this sub-analysis was to investigate the associations between the extent of PA and body composition parameters, adherence to the MeDi, and glycemic control in Dalmatian T2DM patients.

## 2. Materials and Methods

### 2.1. Study Design

This cross-sectional study was conducted at the Division of Nephrology, Dialysis and Arterial Hypertension, Department of Internal Medicine, University Hospital Split and the Division of Medical Laboratory Diagnostic, University Hospital of Split during World Diabetes Day 2023, between November and December 2023, as part of a public appeal for diabetics. The Ethics Committee of the University Hospital of Split accepted the study protocol on 27 November 2023 (Number: 2181-96147/01/06/LJ.7.-23-02, Class: 500-03/23-01/225). All participants provided written consent after being informed about the nature and purpose of the study. This study takes a closer look at PA as part of a sub-analysis, expanding on the results from a previously published paper [18].

### 2.2. Population

**Figure 1:**
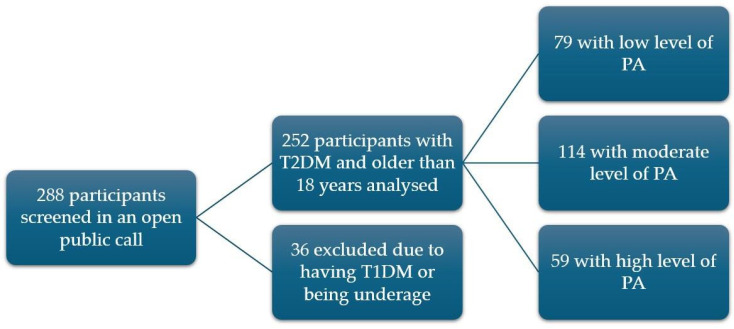
Study participant selection protocol. Abbreviations: T2DM—type 2 diabetes mellitus, T1DM—type 1 diabetes mellitus, PA—physical activity.

During this public call, 288 participants were screened. The inclusion criteria required participants to have an existing diagnosis of T2DM and to be over 18 years of age. The exclusion criteria included a diagnosis of type 1 diabetes mellitus (T1DM). Of the original group, 36 were excluded because they had T1DM, with 31 over 18 years old and 5 under 18 years old. Finally, a total of 252 participants (122 men and 130 women) with T2DM were included in the analysis. In addition, the participants were divided into groups according to the level of their average PA, estimated via IPAQ-SF, and analyzed as such. On average, 79 participants had a low PA level, 114 had a moderate PA level, and 59 had a high PA level. According to the criteria set by the American Diabetes Association, all participants have previously been diagnosed with T2DM by their family doctor or endocrinologist [19]. A detailed study participant selection is shown in Figure 1.

To collect relevant medical information for this study, the participants’ medical records were inspected, and detailed information about their personal history was collected in direct interviews. Additionally, each participant underwent measurements of relevant anthropometric features, including a body mass composition measurement, a muscle strength assessment, a blood pressure measurement, and blood and urine samples for laboratory analysis.

### 2.3. Body Composition, Anthropometric, Muscle Strength, and Blood Pressure Measurements

The MC-780 Multi-frequency Segmental Body Mass Analyzer (Tanita, Tokyo, Japan) was used to determine each participant’s body composition with bioelectrical impedance analysis technology [20]. The body mass (kg), fat-free mass (FFM; kg), visceral fat (VF) level, percentage of muscle mass (PMM; %), fat mass (FM; % and kg), extracellular water (ECW; kg), intracellular water (ICW; kg), total body water (TBW; kg), and phase angle (PhA; °) are among the body composition characteristics estimated by this method. Participants with an implanted pacemaker or cardioverter defibrillator or participants who had a limb amputated were not included in this measurement due to the potential for interference with the devices and the inability to calibrate in the event of limb loss. Height was measured using a stadiometer while circumferences of the waist (WC), hip (HC), and mid-upper arm (MUAC) were measured using the non-stretchable, flexible measuring tape. Body mass index (BMI) and the waist-to-hip ratio (WHR) were calculated from all the parameters above mentioned.

An Omron M6 Comfort HEM-7360-E digital sphygmomanometer (Omron, Kyoto, Japan) was used to obtain peripheral blood pressure readings. From three measurements at one-minute intervals, the average of the last two readings was taken to obtain the peripheral systolic and diastolic blood pressure (pSBP and pDBP, respectively) data.

### 2.4. Mediterranean Diet Serving Score

Adherence to MeDi was evaluated using the validated Mediterranean Diet Serving Score (MDSS) questionnaire. This tool accounts for the intake of 14 distinct foods and food groups, with frequency measured per meal, day, or week. MeDi recommendations for which points were awarded are a daily intake of vegetables, fruits, whole grains, dairy, olive oil, and wine (1–2 glasses daily depending on the sex), weekly intake of fish, poultry, potatoes, legumes, eggs, and nuts, and limited weekly intake of red meat and sweets. The highest possible score is 24, with a score of ≥13.50 representing optimal adherence to the MeDi [21].

### 2.5. Assessment of Physical Activity

The previously translated Croatian version of the IPAQ-SF [21] was used to assess the level of PA [22]. The IPAQ-SF addresses the number of days and time spent in moderate intensity, vigorous intensity, and walking of at least 10 min duration the last 7 days, and includes time spent sitting on weekdays the last 7 days. The IPAQ-SF sum score is expressed in metabolic equivalent of task (MET)—minutes per day or week. In the present study, data processing and analysis were calculated according to the official IPAQ-SF scoring protocol. MET minutes represent the amount of energy expended carrying out PA. A MET is a multiple of patients’ estimated resting energy expenditure. One MET is what a person expends when at rest. Therefore, 2 METs are twice what a person expends at rest. To obtain a continuous variable score from the IPAQ-SF (MET minutes a week), we considered walking to be 3.3 METs, moderate-intensity PA to be 4 METs, and vigorous-intensity PA to be 8 METs.

Furthermore, to classify a high level of PA, participants had to have a vigorous-intensity activity on at least 3 days achieving a minimum total PA of at least 1500 MET minutes a week, or any combination of walking, moderate-intensity, or vigorous-intensity activities on 7 or more days while achieving a minimum total PA of at least 3000 MET minutes a week. For a moderate level of PA, participants had to have at least 3 days of vigorous-intensity activity and/or walking of at least 30 min per day, moderate-intensity activity and/or walking of at least 30 min per day on 5 or more days, or any combination of walking, moderate, or vigorous intensity activities on 5 or more days, achieving a minimum total PA of at least 600 MET minutes a week. If none of the listed criteria were met by the participants, it was classified as a low level of PA. Both classification of low, moderate, or high levels of PA, and individual total MET score as well as IPAQ-SF categories, were analyzed in this study.

### 2.6. Lifestyle Questionnaire, Medical History, and Clinical and Laboratory Parameters

A team of physicians, medical students, and dietitians presented the lifestyle questionnaire, which included questions about general participant information, medical history, and eating habits while being supervised by certified medical professionals. The questionnaire gathered essential information such as age, gender, prescribed medications, length of T2DM therapy, and smoking habits (including past smoking history and duration for current smokers). Participants were also queried about their medical history, such as whether they had ever seen a nephrologist and/or endocrinologist, and if they ever received any nutritional advice. Furthermore, information on all relevant comorbidities such as arterial hypertension (AH), KD, cerebrovascular disease (CBD), cardiovascular disease (CVD), and other chronic diseases was collected.

The blood and morning urine samples were assessed for the following, using methods described previously [18]. The collected data included the following laboratory parameters: red blood cell count (RBC; ×10^12^/L), hemoglobin (Hb; g/L), mean corpuscular volume (MCV; fL), mean cellular hemoglobin concentration (MCHC; g/L), mean cellular hemoglobin (MCH; pg), hematocrit (Htc; L/L), red cell distribution width (RDW; %), platelet count (×10^9^/L), white blood cell count (WBC; ×10^9^/L), neutrophils (×10^9^, %), monocytes (×10^9^, %), lymphocytes (×10^9^, %), basophiles (×10^9^, %), eosinophiles (×10^9^, %), glucose (mmol/L), hemoglobin A1c (HbA1c; %), triglycerides (Tg; mmol/L), high-density lipoprotein cholesterol (HDL; mmol/L), total cholesterol (mmol/L), low-density lipoprotein cholesterol (LDL; mmol/L), creatinine (μmol/L), creatinine in urine (mmol/L), albuminuria (mg/L), estimated glomerular filtration rate (eGFR) using Chronic Kidney Disease Epidemiology Collaboration CKD-EPI (mL/min/1.73 m^2^), and urine albumin-to-creatinine ratio (ACR; mg/mmol).

### 2.7. Statistical Analysis

Categorical data are presented with absolute and relative frequencies. Differences in categorical variables were tested using the chi-square test. The normality of the distribution of numerical variables was tested using the Shapiro–Wilk test, and since the distribution does not follow a normal one, the data are described using the median and interquartile range boundaries. To test differences in continuous variables between two independent groups, the Mann–Whitney U test was used, and for three or more groups, the Kruskal–Wallis test (with the Conover post hoc test). The strength of associations is given by Spearman’s correlation coefficient (Rho). Regression analysis (with adjustments) was used to test which predictors significantly affect the outcomes. All *p*-values are two-sided. The significance level was set at α (alpha) = 0.05. Data analysis was performed using MedCalc^®^ Statistical Software version 22.023 [23] and SPSS ver. 23 [24].

## 3. Results

The study sample consisted of 252 T2DM participants, comprising 130 (51.6%) women and 122 (48.4%) men, with a median age of 67 years (60–73). In this sub-analysis, we explored differences in all measured parameters regarding the level of PA. Participants were further categorized into three groups based on their level of PA: low level of PA (79, 31.4%), moderate level of PA (114, 45.2%), and high level of PA (59, 23.4%). The groups were well matched in terms of all demographic and clinical characteristics, except for the number of cardiovascular comorbidities, which were statistically significantly higher in the moderate level PA group compared to low or high level PA groups (*p* = 0.006 for both, respectively). Detailed demographic and clinical data, including age, sex, blood pressure, visits to an endocrinologist and nephrologist, smoking status, pharmacotherapy, and comorbidities, are presented in Table 1.

### 3.1. Laboratory Parameters and Glycemic Control in Relation to Physical Activity Levels

Statistically significant differences between the groups were observed only in total cholesterol, LDL cholesterol, platelet count, and eGFR. Interestingly, higher values of total and LDL cholesterol values were present in a high-level PA group compared to a low- or moderate-level PA group (*p* = 0.02 for both, respectively). All statistically significant laboratory findings, as well as differences in glucose and HbA1c parameters, are presented in Table 2. Other laboratory comparisons, which did not reveal significant differences, are presented in the complete laboratory analysis depiction in Supplementary Table S1.

A positive correlation between eGFR values and total MET score (r = 0.168; *p* = 0.010), number of days with vigorous activity (r = 0.228; *p* < 0.001), and number of minutes on average spent in vigorous activity per day (r = 0.233; *p* < 0.001) was found. Other statistically significant correlations are listed in Table 3, while a full analysis of laboratory parameters correlations is listed in Supplementary Table S2.

### 3.2. Anthropometric and Body Composition Measurements in Relation to Physical Activity Levels

In terms of anthropometric and body composition parameters, only BMI, HC, FM (kg), FM (%), and PhA showed statistically significant differences between groups. Participants in the low-level PA group had a significantly higher BMI (29 kg/m^2^) compared to the moderate- and high-level PA groups (26.9 and 27.3 kg/m^2^, *p* = 0.007, respectively). HC was significantly smaller in the moderate-level PA group compared to the low-level PA group [105 cm (100–112) vs. 109 cm (103–115); *p* = 0.040]. Furthermore, those in a low-level PA group had higher values of FM (kg and % both) compared to moderate- and high-level PA groups (*p* = 0.02 for both, respectively). Participants in the moderate-level PA group had the highest PhA values [5.2 (4.6–5.7)] compared to the other groups (*p* = 0.010). All statistically significant data are shown in Table 4 while the complete analysis is shown in Supplementary Table S3.

There was a negative correlation between the WHR and walking duration in the average walking sessions per day (r= −0.181; *p* < 0.001). A positive correlation was found between PhA and the number of days with vigorous activity (r = 0.186, *p* < 0.001) as well as the number of minutes on average spent in vigorous activity per day (r = 0.162, *p* = 0.010). Moreover, a statistically significant negative correlation was observed between the overall MET score and FM percentage (r = −0.140; *p* = 0.030) and VF level (r = −0.137; *p* = 0.040). Statistically significant correlations are presented in Table 5, while a full analysis is presented in Supplementary Table S4.

### 3.3. Mediterranean Diet Adherence in Relation to Physical Activity Levels

**Figure 2:**
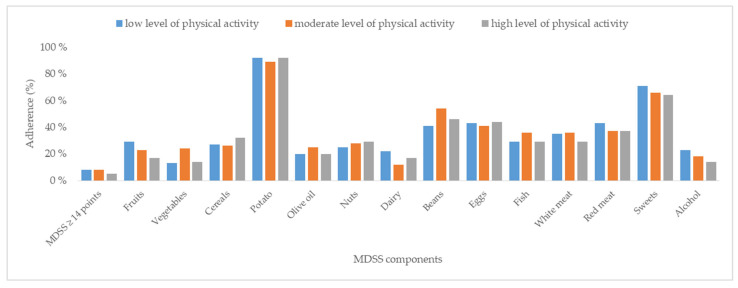
Adherence to the Mediterranean diet in different levels of physical activity. Abbreviation: MDSS—Mediterranean Diet Serving Score questionnaire.

Regarding adherence to the MeDi, there were no statistically significant differences between the groups (*p* = 0.620) based on the MDSS, with median scores of 7 (5–9) in the low level of PA group, 7 (5–10) in the moderate level of PA group, and 6 (4–9) in the high level of PA group. Additionally, the proportion of participants with low adherence to MeDi principles was similarly low across all groups, with only 8%, 8%, and 5% adherence rates in the low, moderate, and high level of PA groups, respectively (*p* = 0.78). No significant differences were observed in adherence to individual components of the MDSS questionnaire among the groups. All data is depicted in Figure 2. while complete data is shown in Supplementary Table S5.

Exploring the correlations between MDSS components and an overall score and IPAQ-SF categories, the total MDSS had a negative correlation with a number of minutes on average spent in moderate activity per day (r = −0.153, *p* = 0.02), while the total MET score correlated negatively with fresh fruit adherence (r = −0.136, *p* = 0.03). Interestingly, vegetable adherence correlated negatively with the number of minutes on average spent in vigorous activity per day (r = −0.135, *p* = 0.03), and the number of minutes on average spent in moderate activity per day (r = −0.146, *p* = 0.02). Statistically significant correlations are presented in Table 6, while the complete correlation data are presented in Supplementary Table S6.

### 3.4. Regression Analysis

To further identify independent predictors of BMI, FM (kg), HbA1c, FFM, and total MET value, a multivariance linear regression was performed. The analysis for HbA1c, total MET, FFM, and FM (kg) was adjusted for gender, age, and BMI, while the analysis for BMI was adjusted for gender and age.

Neutrophils and triglycerides were found as a statistically significant positive predictor of HbA1c. Furthermore, FM (kg), TBW, VF level, and PhA were identified as positive predictors, while FFM, ICM, and FM (%) were identified as negative predictors of BMI values. Only eGFR was found as a significant positive predictor for total MET score. When observing predictors for VF level, FFM, hyperlipidemia, MCH, and pSBP were found to be positive predictors, while TBW, eGFR, and pDBP were found as negative predictors. In an analysis for FFM, TBW, ECW, VF level, and RBC were found as positive predictors of FFM values, while only ICW was found as a negative predictor. Finally, for FM (kg), TBW and PhA were identified as positive predictors, while ICW was determined as a negative predictor of FM (kg) values. All data and analysis are presented in Table 7.

## 4. Discussion

The baseline population in our study was largely obese and had low adherence to MeDi. As previously stated, this cohort also had little awareness of their KD. However, in terms of PA, people living with DM in Dalmatia appear to be reasonably well informed, considering that only 31% of the participants were exposed to low levels of PA [18].

### 4.1. Clinical and Laboratory Parameters and Physical Activity

In our cohort, the glycemic control determined by HbA1c does not seem to be associated with the level of PA, which is in contrast with the current evidence [7,25]. Several trials found a combination of aerobic and resistance training to be superior to aerobic or resistance training alone at lowering HbA1c levels [26,27]. Combined exercise training improved blood glucose fluctuations as well [28]. Recently, the STRONG-D trial showed that resistance training alone was effective and superior to aerobic training alone for reducing HbA1c levels in individuals with normal-weight T2DM, with no significant difference observed between resistance training alone and combination training [2]. Overall, there were no significant differences in long-term glycemic control between prescribed intensities and volumes of resistance training [29]. Therefore, regular exercise is beneficial in T2DM, independent of weight loss, but concurrent weight reduction plays a contributory role [10,30].

Another significant finding is the link between eGFR and the level of PA. Pengfei et al. discovered a strong association between kidney function indicators and various intensities of PA among diabetic individuals progressing to chronic KD. According to this study, such benefits were most obvious at moderate levels of PA, which included light workouts with a slight impact on heart rate variability. The study further proved that 35 h per week was the maximum amount of time one may engage in these forms of exercise, and patients should not go beyond that to avoid detrimental impacts [31].

Unexpectedly, participants in our study who had higher levels of PA also had higher levels of total cholesterol and LDL, which contradicts prior evidence showing regular PA reduces Tg, total cholesterol, and LDL in diabetic patients [32]. This disparity in our studied sample can be attributed in part to poor dietary habits as seen by low MeDi adherence. It is important to emphasize that 45% of diabetics in our study have hyperlipidemia, yet only 37% of the overall group is prescribed statins. Unfortunately, we do not have statistics on statin therapy dosage or adherence. As statin adherence and drug dosage have a substantial effect on lipid profile alterations, this low prescription and questionable adherence may imply poor lipid control in this T2DM cohort.

Regarding comorbidities, our study’s discovery of a higher incidence of cardiovascular comorbidities in the moderate PA group of participants defies established beliefs about PA’s protective role in cardiovascular health [33]. As previous studies have shown, morbidity in the general population is higher among residents of deprived areas than in more affluent areas [34]. Furthermore, those with poorer health care available generally had higher morbidity and were more likely to respond to these free public health screening appeals. Therefore, these paradoxical results could be attributed to the participants’ pre-existing cardiovascular issues and the fact that they were advised to engage in moderate PA as a part of a rehabilitation program, rather than prevention.

### 4.2. Body Composition, Anthropometric Parameters, and Physical Activity

In our population, PA seems to be closely related to body composition and anthropometric parameters. FM (kg), TBW, PhA, and VF levels were found as positive predictors for BMI, and FFM, ICW, and FM percentages were found as negative predictors. It appears that BMI in our population represents fat tissue, a concept that is extensively debated [35]. Participants with low levels of PA had a higher BMI compared to those with moderate and high levels of PA, while those with moderate levels of PA had smaller HC and the highest PhA values. Furthermore, positive correlations were observed between PhA and PA levels, while PA was negatively correlated with FM percentage and visceral adipose tissue. This is likely because PA, especially when moderate or intense, can reduce body fat and improve overall body composition, as opposed to diet alone [36,37]. Regular exercise helps preserve lean body mass, which not only contributes to better glucose regulation but also increases resting energy expenditure, thereby protecting against excess fat accumulation [38]. Conversely, those with lower PA levels experience less muscle preservation and higher fat accumulation, which contributes to a higher BMI [39,40]. These findings further explain why BMI was negatively correlated with walking frequency and duration in this study. Additionally, there were negative correlations between BMI and WHR. In general, evidence suggests that diabetes patients have a higher prevalence of increased WHR, which is strongly associated with age, gender, and BMI [41]. According to different research, PA (particularly intense) paired with a low-calorie diet appears to be the most efficient in reducing body fat [36,42]. Most notably, a recent meta-analysis found that exercise efficiently reduces visceral and perhaps liver adipose tissue in obesity-related T2DM [43]. This is an important target tissue since VF increases insulin resistance and is associated with greater mortality in diabetic individuals [44,45].

When observing the modality of PA, multiple studies indicated that a combination of aerobic and strength exercises provided the best results for glycemic control and fat reduction, especially around the abdominal area, in individuals with T2DM [37,46,47]. However, in a large cohort, both the total volume and intensity of PA energy expenditure are independently linked to body fatness, with higher intensity PA contributing to lower body fat, but overall volume has a stronger influence [40]. Overall, these findings reinforce current PA guidelines, which state that rather than focusing on specific intensity or duration goals, any movement is beneficial [48]. Therefore, increasing PA levels, particularly through moderate to intense exercise, seems critical in improving body composition and managing diabetes-related health risks.

Interestingly, significant predictors of FM were neither PA nor MeDi adherence in our studied population. The possible explanation regarding the MeDi could be really low adherence to that dietary pattern among Dalmatian T2DM participants. As MeDi refers to a diet that emphasizes plants, consumption of extra virgin olive oil, fish and seafood, dairy and poultry and a limited amount of red meat, processed foods, and sugar it follows the “MeDi Pyramid” which portions foods by frequency, balance macronutrients and has moderate calorie restriction. It includes lifestyle factors such as physical activity, hydration, and socializing, which are embedded in the holistic diet and culture form [49].

MeDi and PA are two types of lifestyle interventions that have been shown to be effective against T2DM and obesity [50]. MeDi consists mainly of plant-based foods with higher levels of unsaturated fatty acids, which help control blood glucose levels and insulin sensitivity and also reduce the development of T2DM by reducing inflammation and oxidative stress. Due to the fiber it contains, it has satiating properties and also helps control weight [51,52]. Similarly, insulin sensitivity improves with regular and moderate to intense PA, blood glucose control, and body composition, which can be carried out efficiently and with minimal cardiovascular risk, and also leads to positive muscle mass maintenance [7,53,54,55,56].

The 12-month weight loss lifestyle intervention, which was based on energy-restricted MeDi and PA promotion, successfully reduced cardiovascular risk factors and adiposity and improved glycemic control, insulin sensitivity, and dyslipidemia in participants with or at risk for T2DM, according to the PREDIMED-Plus trial, which involved more than 600 overweight/obese patients with metabolic syndrome [57]. Obesity is a multifaceted condition having physical aspects in terms of elevated CVD risk, the burden of malignancies associated with obesity, and multiple gastrointestinal, musculoskeletal, respiratory, and reproductive health impacts [58]. It also adds greatly to the burden of metabolic changes by exacerbating the dysfunction of key metabolic organs, including the liver, muscle, and pancreas. The ectopic fat accumulates in the non-adipose tissues, further worsening their functioning and enhancing insulin resistance. The release of excess free fatty acids—and beyond that—of the inflammatory mediators by adipocytes disrupts normal cellular processes, creating a vicious cycle of metabolic dysfunction [59]. Psychological aspects of obesity are also important since obesity significantly affects the quality of life of those affected, as many of them are subject to increased stigmatization and discrimination, which can contribute to the development or worsening of mental health problems such as depression, anxiety, eating disorders and substance abuse [60].

Given all mentioned, implementing lifestyle changes through improved PA and adherence to a balanced MeDi should be the cornerstone of lifestyle management of T2DM and more effort should be put in that direction in the Dalmatian population.

### 4.3. Mediterranean Diet and Physical Activity

Regarding adherence to the MeDi, there were no statistically significant differences across PA groups in our study population. Contrary, a systematic review and meta-analysis of the relationships between adherence to the MeDi and cardiorespiratory, motor, and musculoskeletal fitness in adults found that greater adherence to the MeDi was significantly positively associated with higher levels of physical fitness, particularly cardiorespiratory and musculoskeletal fitness in the cross-sectional studies among adults of all ages. Prospective studies demonstrated superior musculoskeletal and total fitness amongst older adults with greater adherence to MeDi [61].

### 4.4. Limitations

The cross-sectional design of this study is preventing us from drawing causal conclusions. The level of PA was self-reported by respondents, which may lead to recall bias, and the sample size limits the generalizability of our results. Future research should investigate the causality of the observed associations, particularly the relationship between moderate PA and cardiovascular comorbidities in a larger population of T2DM participants.

## 5. Conclusions

The synergistic effect of MeDi adherence and PA is crucial in managing T2DM, yet our study underscores the low adherence to MeDi within our cohort. Our results point out that although PA positively correlates with improved body composition and metabolic variables, inadequate adherence to MeDi can restrict these benefits. This lack of adherence to dietary recommendations, combined with considerable variability in PA intensities and modalities, suggests that both should be improved for optimal health outcomes. Furthermore, aerobic fitness alone is insufficient. Both resistance training and adherence to MeDi should be strongly encouraged for optimal glucose balance outcomes. As a result, all T2DM-related public health campaigns and initiatives must strongly promote MeDi and regular mixed PA modalities.

## Figures and Tables

**Table 1:** General characteristics, pharmacotherapy, and comorbidities of studied population.

General Data		Low Level PA (n = 79)	Moderate Level PA (n = 114)	High Level PA (n = 59)	Total (n = 252)	*p*
Age (years), median (IQR)		66 (60–72)	68 (60–74)	68 (62–74)	67 (60–73)	0.59 ^†^
Sex, n (%)						
Men		38 (48)	58 (51)	26 (44)	122 (48)	0.70 *
Women		41 (52)	56 (49)	33 (56)	130 (52)	
pSBP ^1^ (mmHg), median (IQR)		138 (122–149)	138 (127–153)	144 (128–151)	138 (125–152)	0.55 ^†^
pDBP ^1^ (mmHg), median (IQR)		80 (74–87)	82 (76–88)	85 (77–91)	81 (75–88)	0.14 ^†^
Endocrinologist examination, n (%)		51 (65)	87 (76)	40 (68)	178 (71)	0.18 *
Nephrologist examination, n (%)		22 (28)	40 (35)	19 (32)	81 (32)	0.57 *
Regular nephrologist examination, n (%)		9 (11)	15 (13)	8 (14)	32 (13)	0.91 *
Smoking, n (%)		13 (16)	20 (18)	11 (19)	44 (17)	0.95 *
Duration of smoking (years), median (IQR)		40 (28–44)	30 (21–45)	30 (25–40)	30 (25–44)	0.77 ^†^
Former smokers, n (%)		26 (33)	43 (38)	18 (31)	87 (35)	0.60 *
Time without smoking (years), median (IQR)		15 (5–30)	19 (10–24)	15 (5–25)	15 (8–25)	0.69 ^†^
Insulin, n (%)		16 (22)	36 (32)	18 (31)	70 (29)	0.28 *
Metformin, n (%)		55 (74)	72 (64)	37 (63)	164 (67)	0.27 *
SGLT2 inhibitors, n (%)		12 (16)	22 (20)	11 (19)	45 (18)	0.84 *
GLP-1 receptor agonists, n (%)		8 (11)	4 (4)	2 (3)	14 (6)	0.10 *
DPP-4 inhibitors, n (%)		19 (26)	28 (25)	13 (22)	60 (24)	0.88 *
Sulfonamides, n (%)		9 (12)	16 (14)	7 (12)	32 (13)	0.87 *
Thiazolidinediones, n (%)		4 (5)	2 (2)	5 (8)	11 (4)	0.10 *
Alfa-glucosidase inhibitors, n (%)		3 (4)	3 (3)	3 (5)	9 (4)	0.69 *
Statins, n (%)		30 (38)	41 (36)	22 (37)	93 (37)	0.97 *
Statin types (n = 86)	Atorvastatin, n (%)	17 (68)	25 (63)	9 (43)	51 (59)	0.35 *
	Rosuvastatin, n (%)	7 (28)	14 (35)	10 (48)	31 (36)	
	Simvastatin, n (%)	1 (4)	1 (3)	2 (10)	4 (5)	
KD ^1^ (per anamnesis), n (%)		4 (5)	9 (8)	3 (5)	16 (6)	0.66 *
AH ^1^, n (%)		35 (44)	48 (42)	29 (49)	112 (44)	0.68 *
Hyperlipidemia, n (%)		37 (47)	51 (45)	25 (42)	113 (45)	0.87 *
Inflammatory rheumatic disease, n (%)		11 (14)	19 (17)	8 (14)	38 (15)	0.81 *
CBD ^1^, n (%)		2 (3)	3 (3)	2 (3)	7 (3)	>0.99 *
CVD ^1^, n (%)		14 (18)	40 (35)	10 (17)	64 (25)	0.006 *
Malignancies, n (%)		6 (8)	8 (7)	4 (7)	18 (7)	0.98 *
PAD ^1^, n (%)		3 (4)	6 (5)	2 (3)	11 (4)	0.86 *
Depression, n (%)		0	6 (5)	2 (3)	8 (3)	0.09 *
Hypothyroidism, n (%)		7 (9)	12 (11)	3 (5)	22 (9)	0.49 *

* χ^2^ test; ^†^ Kruskal–Wallis test. ^1^ Abbreviations: pSBP—peripheral systolic blood pressure, pDBP—peripheral diastolic blood pressure, SGLT2—sodium/glucose cotransporter 2, GLP-1—glucagon-like peptide-1, DPP-4—dipeptidyl peptidase-4, KD—kidney disease, AH—arterial hypertension, CBD—cerebrovascular disease, CVD—cardiovascular disease, PAD—peripheral artery disease.

**Table 2:** Differences in laboratory parameters regarding the level of physical activity (only statistically significant along with glucose and HbA1c).

	Low-Level PA (n = 79)	Moderate-Level PA (n = 114)	High-Level PA (n = 59)	Total (n = 252)	*p* *
Glucose (mmol/L), median (IQR)	6.4 (5.3–9)	7.1 (5.8–9.2)	6.9 (5.6–8.8)	6.9 (5.7–9.08)	0.13
HbA1c ^1^ (%), median (IQR)	6.5 (6–7.1)	6.8 (6.3–7.4)	6.5 (6.1–7.3)	6.7 (6.1–7.3)	0.15
Platelet count (×10^9^/L), median (IQR)	252 (215–281)	218 (188–272)	242 (208–282)	238 (198.25–280.5)	0.04 ^†^
Cholesterol (mmol/L), median (IQR)	4.40 (3.7–5.4)	4.45 (3.7–5.4)	5 (4.5–5.9)	4.6 (3.8–5.48)	0.02
LDL ^1^ cholesterol (mmol/L), median (IQR)	2.1 (1.8–3)	2.2 (1.6–3)	2.6 (2.3–3.4)	2.4 (1.8–3.1)	0.02
eGFR CKD-EPI ^1^ (mL/min/1.73 m^2^)					
≥90	25 (32)	21 (18)	24 (41)	70 (28)	0.001
60–89	33 (42)	73 (64)	29 (49)	135 (54)	
45–59	6 (8)	12 (11)	5 (8)	23 (9)	
30–44	13 (16)	7 (6)	1 (2)	21 (8)	
15–29	2 (3)	1 (1)	0	3 (1)	

* Kruskal–Wallis test (Conover post hoc test), ^†^ low-level versus moderate-level PA. ^1^ Abbreviations: PA—physical activity, HbA1c—hemoglobin A1c, LDL—low-density lipoprotein, eGFR CKD-EPI—estimated glomerular filtration ratio using Chronic Kidney Disease Epidemiology Collaboration.

**Table 3:** Statistically significant correlations of laboratory parameters (along with glucose and HbA1c) with IPAQ-SF categories.

	IPAQ1 ^1^	IPAQ2 ^1^	IPAQ3 ^1^	IPAQ4 ^1^	IPAQ5 ^1^	IPAQ6 ^1^	IPAQ7 ^1^	Total MET ^1^ Score
RBC ^1^ (×10^12^/L)	0.146 (0.02)	0.123 (0.05)	−0.014 (0.82)	0.001 (>0.99)	0.035 (0.58)	0.077 (0.22)	−0.027 (0.67)	0.110 (0.08)
MCV ^1^ (fL)	−0.058 (0.36)	−0.062 (0.33)	−0.026 (0.68)	−0.016 (0.80)	0.092 (0.15)	0.066 (0.30)	−0.144 (0.02)	0.023 (0.72)
MCH ^1^ (pg)	0.001 (0.99)	−0.015 (0.82)	−0.024 (0.70)	−0.031 (0.62)	0.075 (0.24)	0.115 (0.07)	−0.158 (0.01)	0.050 (0.43)
MCHC ^1^ (g/L)	0.084 (0.19)	0.069 (0.27)	0.012 (0.85)	−0.027 (0.67)	0.013 (0.83)	0.132 (0.04)	−0.069 (0.27)	0.065 (0.31)
RDW ^1^ (%)	−0.129 (0.04)	−0.110 (0.08)	−0.060 (0.34)	−0.071 (0.26)	0.031 (0.62)	−0.175 (0.01)	0.069 (0.27)	−0.124 (0.05)
Thrombocytes (×10^9^/L)	0.090 (0.16)	0.061 (0.33)	0.057 (0.36)	0.028 (0.66)	−0.141 (0.03)	−0.204 (<0.001)	0.085 (0.18)	−0.071 (0.26)
Neutrophils (%)	−0.067 (0.29)	−0.089 (0.16)	−0.109 (0.09)	−0.130 (0.04)	−0.001 (0.99)	0.030 (0.64)	−0.034 (0.60)	−0.026 (0.68)
Basophils (%)	0.011 (0.86)	−0.016 (0.79)	0.138 (0.03)	0.095 (0.13)	0.039 (0.54)	−0.035 (0.58)	−0.014 (0.83)	0.015 (0.81)
Neutrophils (×10^9^/L)	−0.033 (0.60)	−0.077 (0.22)	−0.116 (0.07)	−0.124 (0.05)	−0.058 (0.36)	−0.015 (0.81)	−0.044 (0.49)	−0.045 (0.48)
eGFR CKD-EPI ^1^ (mL/min/1.73 m^2^)	0.228 (<0.001)	0.233 (<0.001)	0.037 (0.56)	0.086 (0.17)	−0.034 (0.59)	0.116 (0.07)	−0.085 (0.18)	0.168 (0.01)
Cholesterol (mmol/L)	0.125 (0.05)	0.142 (0.02)	0.143 (0.02)	0.107 (0.09)	0.036 (0.57)	0.009 (0.89)	−0.076 (0.23)	0.116 (0.07)
Tg ^1^ (mmol/L)	−0.003 (0.97)	0.016 (0.81)	−0.076 (0.23)	−0.084 (0.18)	−0.113 (0.07)	−0.146 (0.02)	0.021 (0.74)	−0.080 (0.20)
HDL ^1^ cholesterol (mmol/L)	−0.032 (0.61)	0.011 (0.87)	0.097 (0.12)	0.059 (0.35)	0.123 (0.05)	0.030 (0.64)	−0.059 (0.35)	0.032 (0.62)
LDL ^1^ cholesterol (mmol/L)	0.135 (0.03)	0.136 (0.03)	0.148 (0.02)	0.133 (0.03)	0.020 (0.75)	0.027 (0.66)	−0.068 (0.28)	0.121 (0.06)
Glucose (mmol/L)	0.019 (0.76)	0.013 (0.84)	−0.028 (0.66)	0.016 (0.80)	0.026 (0.68)	0.005 (0.94)	−0.048 (0.45)	0.067 (0.29)
HbA1c ^1^ (%)	0.029 (0.65)	0.026 (0.69)	−0.090 (0.16)	−0.072 (0.26)	0.052 (0.41)	0.039 (0.54)	−0.100 (0.21)	0.023 (0.71)
Creatinine (mmol/L) (from urine sample)	0.186 (<0.001)	0.168 (0.01)	−0.052 (0.41)	0.017 (0.79)	−0.019 (0.76)	0.194 (<0.001)	0.054 (0.39)	0.185 (<0.001)

Data format: Rho (*p*-value). ^1^ Abbreviations: IPAQ1—number of days with vigorous activity, IPAQ2—number of minutes on average spent in vigorous activity per day, IPAQ3—number of days with moderate activity, IPAQ4—number of minutes on average spent in moderate activity per day, IPAQ5—number of days with continuous walking activity, IPAQ6—number of minutes on average spent walking per day, IPAQ7—time spent sitting per day (in hours), MET—metabolic equivalent of task, RBC—red blood cell count, MCV—mean corpuscular volume, MCH—mean cellular hemoglobin, MCHC—mean cellular hemoglobin concentration, RDW—red cell distribution width, eGFR CKD-EPI—estimated glomerular filtration ratio using Chronic Kidney Disease Epidemiology Collaboration, Tg—triglycerides, HDL—high-density lipoprotein, LDL—low-density lipoprotein, HbA1c—hemoglobin A1c.

**Table 4:** Statistically significant anthropometric and body composition measurements regarding the level of physical activity.

Anthropometric and Body Composition Measures	Low-Level PA (n = 79)	Moderate-Level PA (n = 114)	High-Level PA (n = 59)	Total (n = 252)	*p* *
BMI ^1^ (kg/m^2^), median (IQR)	29 (25.35–33.2)	26.9 (23.7–29.8)	27.3 (22.2–31.6)	27.6 (24.2–31)	0.007
HC ^1^ (cm), median (IQR)	109 (103–115)	105 (100–112)	108 (99–113)	107 (100–114)	0.04
FM ^1^ (%), median (IQR)	32.2 (25.65–38.6)	29 (22.8–34.7)	29.3 (22.5–35.6)	30.1 (23.1–36.5)	0.02
FM ^1^ (kg), median (IQR)	26.3 (19.45–36.5)	23.1 (17.4–28.7)	21.7 (16.9–31.9)	23.6 (17.9–31.8)	0.02
PhA ^1^ (°), median (IQR)	5.5 (4.8–6)	5.2 (4.6–5.7)	5.6 (5.08–6.3)	5.4 (4.8–5.9)	0.01

* Kruskal–Wallis test (Conover post hoc test). ^1^ Abbreviations: PA—physical activity, BMI—body mass index, HC—hip circumference, FM—fat mass, PhA—phase angle.

**Table 5:** Statistically significant correlations of anthropometric and body composition measurements with IPAQ-SF categories.

	IPAQ1 ^1^	IPAQ2 ^1^	IPAQ5 ^1^	IPAQ6 ^1^	IPAQ7 ^1^	Total MET ^1^ Score
BMI ^1^ (kg/m^2^)	−0.026 (0.69)	−0.039 (0.54)	−0.257 (<0.001)	−0.103 (0.11)	0.003 (0.96)	−0.087 (0.17)
WHR ^1^	−0.060 (0.35)	−0.056 (0.38)	−0.049 (0.44)	−0.181 (<0.001)	−0.043 (0.50)	−0.088 (0.17)
FM ^1^ (%)	−0.108 (0.09)	−0.091 (0.15)	−0.177 (0.01)	−0.158 (0.01)	−0.021 (0.74)	−0.140 (0.03)
FM ^1^ (kg)	−0.056 (0.38)	−0.065 (0.31)	−0.211 (<0.001)	−0.110 (0.09)	−0.015 (0.82)	−0.084 (0.19)
VF ^1^ level	−0.081 (0.22)	−0.100 (0.13)	−0.163 (0.01)	−0.123 (0.06)	0.038 (0.56)	−0.137 (0.04)
ECW ^1^ (kg)	0.039 (0.55)	0.003 (0.96)	−0.146 (0.02)	0.010 (0.88)	0.075 (0.24)	0.004 (0.95)
PhA ^1^ (°)	0.186 (<0.001)	0.161 (0.01)	−0.039 (0.55)	0.077 (0.24)	0.017 (0.80)	0.113 (0.09)

Data format: Rho (*p*-value). ^1^ Abbreviations: IPAQ1—number of days with vigorous activity, IPAQ2—number of minutes on average spent in vigorous activity per day, IPAQ5—number of days with continuous walking activity, IPAQ6—number of minutes on average spent walking per day, IPAQ7—time spent sitting per day (in hours), MET—metabolic equivalent of task, BMI—body mass index, WHR—waist-to-hip ratio, FM—fat mass, VF—visceral fat, ECW—extracellular water, PhA—phase angle.

**Table 6:** Statistically significant correlations of MDSS components and total Mediterranean diet adherence with IPAQ-SF categories.

	IPAQ2 ^1^	IPAQ4 ^1^	IPAQ6 ^1^	IPAQ7 ^1^	Total MET ^1^ Score
Total MDSS	−0.029 (0.65)	−0.153 (0.02)	−0.037 (0.56)	0.028 (0.67)	−0.092 (0.15)
Grains	0.040 (0.53)	−0.011 (0.86)	−0.065 (0.30)	−0.041 (0.52)	0.041 (0.51)
Potato	0.008 (0.90)	0.065 (0.30)	0.019 (0.77)	0.009 (0.89)	0.045 (0.48)
Olive oil	−0.037 (0.56)	−0.104 (0.10)	−0.167 (0.01)	0.114 (0.07)	−0.076 (0.23)
Nuts	0.045 (0.48)	−0.051 (0.42)	−0.013 (0.84)	−0.029 (0.65)	0.026 (0.68)
Fresh fruit	−0.091 (0.15)	−0.112 (0.08)	−0.122 (0.05)	0.054 (0.39)	−0.136 (0.03)
Vegetables	−0.135 (0.03)	−0.146 (0.02)	−0.021 (0.73)	0.031 (0.63)	−0.116 (0.07)
Dairy	−0.047 (0.46)	−0.042 (0.51)	0.027 (0.67)	0.019 (0.76)	−0.069 (0.27)
Legumes	0.064 (0.31)	0.011 (0.87)	0.100 (0.11)	−0.037 (0.56)	0.095 (0.13)
Eggs	0.152 (0.02)	−0.050 (0.43)	0.001 (0.99)	0.009 (0.89)	0.059 (0.35)
Fish	0.068 (0.28)	−0.010 (0.87)	0.038 (0.55)	−0.159 (0.01)	0.009 (0.88)

Data format: Rho (*p*-value). ^1^ Abbreviations: IPAQ2—number of minutes on average spent in vigorous activity per day, IPAQ4—number of minutes on average spent in moderate activity per day, IPAQ6—number of minutes on average spent walking per day, IPAQ7—time spent sitting per day (in hours), MET—metabolic equivalent of task, MDSS—Mediterranean Diet Serving Score.

**Table 7:** The influence of independent predictors on HbA1c, BMI, fat-free mass, fat mass in kilograms, and total MET value (multivariant linear regression—Stepwise method).

	ß	*p* Value	95% CI	Regression Module
HbA1c *				
Neutrophils (×10^9^/L)	0.138	0.001	0.056–0.220	R = 0.339; R^2^ = 0.115; R^2^_kor_ = 0.096 F_(5, 225)_ = 5.86; *p* < 0.001 Cohen’s f^2^ = 0.10
Tg ^1^ (mmol/L)	0.242	0.001	0.096–0.388	
Constant	6.61	<0.001	5.46–7.76	
**BMI ^†,1^—excluded height and weight from analysis**				
FM ^1^ (kg)	0.464	<0.001	0.369–0.559	R = 0.962; R^2^ = 0.926; R^2^_kor_ = 0.923 F_(9, 221)_ = 305.3; *p* < 0.001 Cohen’s f^2^ = 12.4
TBW ^1^ (kg)	0.922	<0.001	0.714–1.13	
VF ^1^ level	0.325	<0.001	0.249–0.400	
FFM ^1^ (kg)	−0.606	<0.001	−0.761–−0.451	
PhA ^1^ (°)	0.185	<0.001	0.103–0.268	
ICW ^1^ (kg)	−0.059	<0.001	−0.090–−0.027	
FM ^1^ (%)	−0.154	<0.001	−0.270–−0.039	
Constant	13.7	<0.001	9.88–17.43	
Total MET ^1^ score *				
eGFR CKD-EPI ^1^ (mL/min/1.73 m^2^)	43.6	0.007	12.1–75.04	R = 0.036; R^2^ = 0.036; R^2^_kor_ = 0.019 F_(4, 226)_ = 2.13; *p* = 0.07 Cohen’s f^2^ = 0.033
Constant	205.9	0.51	−399.3–800.5	
VF ^1^ level *				
FFM ^1^ (kg)	1.11	<0.001	1.05–1.17	R = 0.944; R^2^ = 0.891; R^2^_kor_ = 0.885 F_(11, 219)_ = 162.2; *p* < 0.001 Cohen’s f^2^ = 8.17
TBW ^1^ (kg)	−1.45	<0.001	−1.53–−1.37	
Hyperlipidemia	0.825	0.003	0.283–1.37	
eGFR CKD-EPI ^1^ (mL/min/1.73 m^2^)	−0.013	0.05	−0.027–0	
MCH (pg)	0.188	0.007	0.051–0.324	
pDBP ^1^ (mmHg)	−0.063	<0.001	−0.094–−0.032	
pSBP ^1^ (mmHg)	0.029	<0.001	0.013–0.045	
Constant	−13.7	<0.001	−19.1–−8.3	
FFM *^,1^—excluded FM (kg) and FM (%) from analysis				
TBW ^1^ (kg)	1.41	<0.001	1.38–1.43	R = 0.994; R^2^ = 0.988; R^2^_kor_ = 0.987 F_(8, 222)_ = 387.8; *p* < 0.001 Cohen’s f^2^ = 66.2
ECW ^1^ (kg)	0.006	<0.001	0.004–0.008	
VF ^1^ level	0.288	<0.001	0.236–0.339	
RBC ^1^ (×10^12^/L)	0.445	0.004	0.147–0.743	
ICW ^1^ (kg)	−0.014	0.05	−0.029–0.0	
Constant	1.73	0.04	−0.01–3.47	
FM ^1^ (kg) *—excluded FFM and FM (%) from analysis				
TBW ^1^ (kg)	−0.376	<0.001	−0.460–0.292	R = 0.901; R^2^ = 0.812; R^2^_kor_ = 0.810 F_(6, 224)_ = 250.2; *p* < 0.001 Cohen’s f^2^ = 0.45
PhA ^1^ (°)	−0.257	0.02	−0.469–−0.045	
ICW ^1^ (kg)	0.091	0.03	0.009–0.174	
Constant	−13.7	<0.001	−18.1–−9.26	

ß—unstandardized regression coefficients. * adjusted for gender, age, BMI. ^†^ adjusted for gender, age. ^1^ Abbreviations: HbA1c—hemoglobin A1c, Tg—triglycerides, BMI—body mass index, FM—fat mass, TBW—total body water, VF—visceral fat, FFM—fat-free mass, PhA—phase angle, ICW—intracellular water, MET—metabolic equivalent of task, eGFR CKD-EPI—estimated glomerular filtration ratio using Chronic Kidney Disease Epidemiology Collaboration, MCH—mean cellular hemoglobin, pSBP—peripheral systolic blood pressure, pDBP—peripheral diastolic blood pressure, ECW—extracellular water, RBC—red blood cell count.

## Data Availability

Data are available upon reasonable request at the corresponding author’s e-mail.

## Supplementary Materials

The following supporting information can be downloaded at: https://www.mdpi.com/article/10.3390/nu17010187/s1, Supplementary Table S1: laboratory parameters of studied population, Supplementary Table S2: correlations of laboratory parameters with IPAQ-SF categories, Supplementary Table S3: anthropometric measurements and body composition in different types of intensity activities, Supplementary Table S4: correlations of anthropometric and body composition measurements with IPAQ-SF categories, Supplementary Table S5: adherence to Mediterranean diet according to levels of physical activity, Supplementary Table S6: correlations of MDSS components and total Mediterranean diet adherence with IPAQ-SF categories.
