# Supplementary material for: The Power of Movement: Linking Physical Activity with Nutritional Health and Blood Sugar Balance in a Dalmatian Type 2 Diabetic Population
Source: Nutrients. 2025 Jan 4;17(1):187. doi: 10.3390/nu17010187 (PMC11722635; doi:10.3390/nu17010187)
Supplement: Supplementary file 1 [file nutrients-17-00187-s001.zip › nutrients-3399363-supplementary.pdf]

Supplementary Table S1. Laboratory parameters of studied population.

	Low level of PA (n=79)	Moderate level of PA (n=114)	High level of PA (n=59)	Total (n=252)	*P**
WBC[1] (x10⁹/L), median (IQR)	6.9 (5.8 - 8)	6.95 (5.95 - 8.5)	6.6 (5.8 - 7.9)	6.8 (5,8 - 8,1)	0.75
RBC[1], median (IQR)	4.62 (4.31 - 4,9)	4.76 (4.39 - 5.1)	4,7 (4.41 - 5)	4.7 (4.37 – 4.99)	0.27
Hb[1] (g/L), median (IQR)	137 (126 - 151)	143 (131 - 152.3)	144 (128 - 152)	141 (128 - 151)	0.14
Htc[1] (L/L), median (IQR)	404 (378 - 432)	417 (384.5 - 446)	420 (382 - 443)	413 (381.25 - 441)	0.13
MCV[1] (fL), median (IQR)	87.9 (85.7 - 90.8)	88.55 (86.2 - 91.3)	88.6 (85.2 - 91)	88.35 (86 - 91)	0.52
MCH[1] (pg), median (IQR)	30 (28.9 - 31.3)	30,3 (29.25 - 31.4)	30.6 (29 - 31.4)	30.3 (29.03 – 31.4)	0.42
MCHC[1] (g/L), median (IQR)	341 (333 - 349)	342 (335 - 348)	340 (337 - 349)	342 (335 – 348.75)	0.62
RDW[1] (%), median (IQR)	12.9 (12.5 - 13.6)	1305 (12.68 - 13.7)	12.8 (12.2 - 13.2)	13 (12.5 – 13.5)	0.06
Platelet count (x10⁹/L), median (IQR)	252 (215 - 281)	218 (188 - 272)	242 (208 - 282)	238 (198.25 – 280.5)	0.04
Neuthrophiles (%), median (IQR)	58.6 (52.4 - 64.9)	60.7 (53.55 - 64,8)	56.2 (52.9 - 66)	59.25 (52.95 – 65.13)	0.34
Lymphocytes (%), median (IQR)	30.2 (23.9 - 35.6)	28.5 (24.95 - 34.9)	30.7 (24.1 - 36.2)	29.2 (24.15 – 35.3)	0.60
Monocytes (%), median (IQR)	7.9 (6.6 - 9.3)	7.85 (6.5 - 8.7)	7.7 (6.6 - 9)	7.85 (6.6 – 8.9)	0.59
Eosinophiles (%), median (IQR)	2.7 (1.5 - 4)	2 (1.4 - 2.9)	2.5 (1.6 - 3.7)	2.2 (1.43 – 3.4)	0.09
Basophiles (%), median (IQR)	0.6 (0.4 - 0.8)	0.6 (0,5 - 0.7)	0.6 (0.5 - 0.9)	0.6 (0.5 – 0.8)	0.87
Glucose (mmol/L), median (IQR)	6.4 (5.3 - 9)	7.1 (5.8 - 9.2)	6.9 (5.6 - 8.8)	6.9 (5.7 – 9.08)	0.13
Creatinine (µmol/L), median (IQR)	81 (64 - 107)	82 (71 - 94.3)	79 (66 - 92)	81 (69 – 96.75)	0.58
eGFR CKD-EPI[1] (mL/min/1.73 m2), median (IQR)	77.7 (57.2 - 94.9)	75.5 (62.6 - 85)	80.3 (66.7 - 94.9)	76.95 (62.48 – 91.18)	0.11
Cholesterol (mmol/L)l, median (IQR)	4.40 (3.7 - 5.4)	4.45 (3.7 - 5.4)	5 (4.5 - 5.9)	4.6 (3.8 – 5.48)	0.02
Tg[1] (mmol/L), median (IQR)	1.5 (1 - 2.1)	1.3 (0.9 - 1.9)	1.4 (0.9 - 2.2)	1.4 (0.9 – 2)	0.47
HDL[1] cholesterol (mmol/L), median (IQR)	1.4 (1.2 - 1.6)	1.5 (1.2 - 1.7)	1.5 (1.2 - 1.7)	1.4 (1.2 – 1.7)	0.49
LDL[1] cholesterol (mmol/L), median (IQR)	2.1 (1.8 - 3)	2.2 (1.6 - 3)	2.6 (2.3 - 3.4)	2.4 (1.8 – 3.1)	0.02
HbA1c[1] (%), median (IQR)	6.5 (6 - 7.1)	6.8 (6.3 - 7.4)	6.5 (6.1 - 7.3)	6.7 (6.1 – 7.3)	0.15
Creatinine (mg/dU), median (IQR)	6.29 (3.9 - 9.5)	7.06 (4.37 - 9.5)	6.99 (4.64 - 10.7)	6.78 (4.44 – 9.61)	0.43
Albuminuria (mg/dU), median (IQR)	5 (2 - 14)	5.5 (2 - 13)	5 (2 - 13)	5 (2 - 13)	>0.99
ACR[1] (mg/mmol), median (IQR)	0.74 (0.43 - 1.9)	0.76 (0.52 - 1.7)	0.69 (0.39 - 1.5)	0.75 (0.46 – 1.73)	0.65
eGFR					
≥ 90	25 (32)	21 (18)	24 (41)	70 (28)	0.001
60 – 89	33 (42)	73 (64)	29 (49)	135 (54)	
45 – 59	6 (8)	12 (11)	5 (8)	23 (9)	
30 – 44	13 (16)	7 (6)	1 (2)	21 (8)	

15 – 29	2 (3)	1 (1)	0	3 (1)	
ACR					
< 3 mg/mmol	65 (82)	94 (82)	49 (83)	208 (83)	
3 – 30 mg/mmol	9 (11)	15 (13)	9 (15)	33 (13)	0.75
> 30 mg/mmol	5 (6)	5 (4)	1 (2)	11 (4)	

*Kruskal Wallisov test (Post hoc test Conover)

[1] Abbreviations: WBC - leukocytes, RBC - red blood cell count, MCV - mean corpuscular volume, Hb - hemoglobin, MCH - mean cellular hemoglobin, MCHC - RDW - red cell distribution width, MPV - mean platelet volume, eGFR CKD-EPI - estimated glomerular filtration ratio using Chronic Kidney Disease Epidemiology Collaboration, Tg - triglycerides, HDL - high density lipoprotein, LDL - low density lipoprotein, HbA1c - hemoglobin A1c, ACR - albumin-to-creatinine ratio

Supplementary Table S2. Correlations of laboratory parameters with IPAQ-SF categories.

	**IPAQ1**[1]	**IPAQ2**[1]	**IPAQ3**[1]	**IPAQ4**[1]	**IPAQ5**[1]	**IPAQ6**[1]	**IPAQ7**[1]	**Total MET**[1] **score**
RBC[1] (x10^{12}/L)	**0.146 (0.02)**	**0.123 (0.05)**	-0.014 (0.82)	0.001 (>0.99)	0.035 (0.58)	0.077 (0.22)	-0.027 (0.67)	0.110 (0.08)
Hb[1] (g/L)	0.125 (0.05)	0.100 (0.11)	-0.052 (0.41)	-0.034 (0.59)	0.065 (0.30)	0.091 (0.15)	-0.109 (0.09)	0.102 (0.10)
MCV[1] (fL)	-0.058 (0.36)	-0.062 (0.33)	-0.026 (0.68)	-0.016 (0.80)	0.092 (0.15)	0.066 (0.30)	**-0.144 (0.02)**	0.023 (0.72)
MCH[1] (pg)	0.001 (0.99)	-0.015 (0.82)	-0.024 (0.70)	-0.031 (0.62)	0.075 (0.24)	0.115 (0.07)	**-0.158 (0.01)**	0.050 (0.43)
MCHC[1] (g/L)	0.084 (0.19)	0.069 (0.27)	0.012 (0.85)	-0.027 (0.67)	0.013 (0.83)	**0.132 (0.04)**	-0.069 (0.27)	0.065 (0.31)
RDW[1] (%)	**-0.129 (0.04)**	-0.110 (0.08)	-0.060 (0.34)	-0.071 (0.26)	0.031 (0.62)	**-0.175 (0.01)**	0.069 (0.27)	**-0.124 (0.05)**
Thrombocytes (x10^{9}/L)	0.090 (0.16)	0.061 (0.33)	0.057 (0.36)	0.028 (0.66)	**-0.141 (0.03)**	**-0.204 (<0.001)**	0.085 (0.18)	-0.071 (0.26)
Neutrophiles (%)	-0.067 (0.29)	-0.089 (0.16)	-0.109 (0.09)	**-0.130 (0.04)**	-0.001 (0.99)	0.030 (0.64)	-0.034 (0.60)	-0.026 (0.68)
Lymphocytes (%)	0.072 (0.26)	0.101 (0.11)	0.086 (0.17)	0.090 (0.15)	0.014 (0.82)	-0.027 (0.67)	0.047 (0.46)	0.032 (0.61)
Monocytes (%)	0.009 (0.89)	0.004 (0.96)	0.011 (0.86)	0.055 (0.39)	0.057 (0.37)	0.002 (0.97)	-0.035 (0.58)	0.028 (0.66)
Eosinophiles (%)	0.003 (0.97)	-0.001 (0.99)	0.072 (0.26)	0.089 (0.16)	-0.026 (0.69)	0.027 (0.67)	0.016 (0.80)	0.004 (0.94)
Basophiles (%)	0.011 (0.86)	-0.016 (0.79)	**0.138 (0.03)**	0.095 (0.13)	0.039 (0.54)	-0.035 (0.58)	-0.014 (0.83)	0.015 (0.81)
Neutrophiles (x10^{9}/L)	-0.033 (0.60)	-0.077 (0.22)	-0.116 (0.07)	**-0.124 (0.05)**	-0.058 (0.36)	-0.015 (0.81)	-0.044 (0.49)	-0.045 (0.48)
Lymphocytes (x10^{9}/L)	0.024 (0.71)	-0.002 (0.97)	0.007 (0.91)	-0.004 (0.95)	-0.104 (0.10)	-0.060 (0.35)	-0.014 (0.83)	-0.041 (0.52)
Monocytes (x10^{9}/L)	-0.036 (0.57)	-0.084 (0.18)	-0.086 (0.18)	-0.059 (0.35)	-0.052 (0.41)	-0.035 (0.58)	-0.068 (0.29)	-0.043 (0.50)

Eosinophiles (x10⁹/L)	-0.023 (0.72)	-0.038 (0.54)	0.025 (0.70)	0.033 (0.60)	-0.074 (0.24)	-0.010 (0.87)	0.016 (0.80)	-0.033 (0.60)
Basophiles (x10⁹/L)	-0.006 (0.93)	-0.057 (0.36)	0.075 (0.24)	0.036 (0.57)	-0.039 (0.54)	-0.088 (0.16)	-0.046 (0.47)	-0.020 (0.75)
Glucose (mmol/L)	0.019 (0.76)	0.013 (0.84)	-0.028 (0.66)	0.016 (0.80)	0.026 (0.68)	0.005 (0.94)	-0.048 (0.45)	0.067 (0.29)
Creatinine (µmol/L)	-0.067 (0.29)	-0.093 (0.14)	-0091 (0.15)	-0.101 (0.11)	0.056 (0.38)	0.013 (0.84)	0.115 (0.07)	-0.052 (0.41)
eGFR CKD-EPI[1] (mL/min/1.73 m²)	**0.228 (<0.001)**	**0.233 (<0.001)**	0.037 (0.56)	0.086 (0.17)	-0.034 (0.59)	0.116 (0.07)	-0.085 (0.18)	**0.168 (0.01)**
Cholesterol (mmol/L)	**0.125 (0.05)**	**0.142 (0.02)**	**0.143 (0.02)**	0.107 (0.09)	0.036 (0.57)	0.009 (0.89)	-0.076 (0.23)	0.116 (0.07)
Tg[1] (mmol/L)	-0.003 (0.97)	0.016 (0.81)	-0.076 (0.23)	-0.084 (0.18)	-0.113 (0.07)	-**0.146 (0.02)**	0.021 (0.74)	-0.080 (0.20)
HDL-cholesterol[1] (mmol/L)	-0.032 (0.61)	0.011 (0.87)	0.097 (0.12)	0.059 (0.35)	**0.123 (0.05)**	0.030 (0.64)	-0.059 (0.35)	0.032 (0.62)
LDL-cholesterol[1] (mmol/L)	**0.135 (0.03)**	**0.136 (0.03)**	**0.148 (0.02)**	**0.133 (0.03)**	0.020 (0.75)	0.027 (0.66)	-0.068 (0.28)	0.121 (0.06)
Glucose (mmol/L)	0.019 (0.76)	0.013 (0.84)	-0.028 (0.66)	0.016 (0.80)	0.026 (0.68)	0.005 (0.94)	-0.048 (0.45)	0.067 (0.29)
HbA1c (%)	0.029 (0.65)	0.026 (0.69)	-0.090 (0.16)	-0.072 (0.26)	0.052 (0.41)	0.039 (0.54)	-0.100 (0.21)	0.023 (0.71)
Creatinine (mmol/L) (from urine sample)	**0.186 (<0.001)**	**0.168 (0.01)**	-0.052 (0.41)	0.017 (0.79)	-0.019 (0.76)	**0.194 (<0.001)**	0.054 (0.39)	**0.185 (<0.001)**
Albuminuria (mg/mmol)	0.029 (0.64)	0.008 (0.89)	-0.061 (0.33)	-0.074 (0.24)	-0.061 (0.33)	0.015 (0.82)	0.024 (0.70)	-0.002 (0.97)
ACR[1] (mg/mmol)	-0.076 (0.23)	-0.083 (0.19)	-0.040 (0.53)	-0.085 (0.18)	-0.055 (0.39)	-0.090 (0.15)	-0.009 (0.88)	-0.110 (0.08)

* Data format: Rho (p-value)

[1] Abbreviations: IPAQ1 – number of days with vigorous activity, IPAQ2 – number of minutes in average spent in vigorous activity per day, IPAQ3 - number of days with moderate activity, IPAQ4 - number of minutes in average spent in moderate activity per day, IPAQ5 - number of days with continuous walking activity, IPAQ6 – number of minutes in average spent walking per day, IPAQ7 – time spent sitting per day (in hours), MET - metabolic equivalent of task, , RBC - red blood cell count, MCV - mean corpuscular volume, Hb - hemoglobin, MCH - mean cellular hemoglobin, MCHC - mean cellular hemoglobin concentration, RDW - red cell distribution width, eGFR CKD-EPI - estimated glomerular filtration ratio using Chronic Kidney Disease Epidemiology Collaboration, Tg - triglycerides, HDL - high density lipoprotein, LDL - low density lipoprotein, ACR – albumin-to-creatinine ratio.

Supplementary Table S3. Anthropometric measurements and body composition in different types of intensity activities.

**Anthropometric measures**	**Low level of PA (n=79)**	**Moderate level of PA (n=114)**	**High level of PA (n=59)**	**Total (n=252)**	***P****
Height (cm), median (IQR)	171 (164.5 - 176.5)	173 (165 - 180)	174 (165 - 179)	173 (165 - 180)	0.26

Weight (kg), median (IQR)	85 (73.85 - 96)	78 (69.4 - 94)	81.1 (65.9 - 94)	81.5 (69.8 - 94.9)	0.14
BMI[1] (kg/m^2), median (IQR)	29 (25.35 - 33.2)	26.9 (23.7 - 29.8)	27.3 (22.2 - 31.6)	27.6 (24.2 - 31)	**0.007**
MUAC[1] (cm), median (IQR)	30 (27.25 - 32.3)	29.75 (27 - 31.4)	30 (27 - 32)	30 (27 - 32)	0.34
WC[1] (cm), median (IQR)	101 (94 - 113)	100 (89 - 110)	98 (90 - 110)	100 (90 - 110)	0.07
HC[1] (cm), median (IQR)	109 (103 - 115)	105 (100 - 112)	108 (99 - 113)	107 (100 - 114)	**0.04**
WHR[1], median (IQR)	0.93 (0.88 - 1)	0.93 (0.87 - 1)	0.94 (0.87 - 1)	0.93 (0.88 - 0.99)	0.67
FM[1] (%), median (IQR)	32.2 (25.65 - 38.6)	29 (22.8 - 34.7)	29.3 (22.5 - 35.6)	30.1 (23.1 – 36.5)	**0.02**
FM[1] (kg), median (IQR)	26.3 (19.45 - 36.5)	23.1 (17.4 - 28.7)	21.7 (16.9 - 31.9)	23.6 (17.9 – 31.8)	**0.02**
PhA[1], median (IQR)	5.5 (4.8 - 6)	5.2 (4.6 - 5.7)	5.6 (5.08 - 6.3)	5.4 (4.8 – 5.9)	**0.01**
BMI <25, *n* (%)	16 (21)	38 (34)	20 (34)	74 (30)	0.10**
25 ≤ BMI < 30, *n* (%)	29 (38)	46 (41)	21 (36)	96 (39)	
BMI ≥ 30, *n* (%)	32 (42)	27 (24)	18 (31)	77 (31)	
VF[1] level, median (IQR)	12 (10 - 15)	11 (8.75 - 14)	11 (8 - 13)	11 (9 - 14)	0.06
FFM[1] (kg), median (IQR)	56.3 (48.95 - 66.9)	56.8 (47.9 - 68.7)	57 (47.9 - 68.2)	56.8 (48.4 – 68.2)	0.95
TBW[1] (kg), median (IQR)	39 (34.65 - 46.6)	39.4 (34.2 - 46.7)	40 (33.8 - 47.2)	39.6 (34.2 – 46.7)	0.23
ECW[1] (kg), median (IQR)	18.4 (16.5 - 21.1)	17.8 (15.2 - 20.3)	18.3 (15.8 - 20.4)	18.1 (15.8 – 20.4)	0.63
ICW[1] (kg), median (IQR)	21.8 (18.95 - 27.2)	21.6 (18.4 - 26.5)	22.1 (18.2 - 27.5)	21.7 (18.6 – 27.1)	0.63
PMM[1] (%), median (IQR)	53.5 (46.45 - 63.6)	53.9 (45.5 - 65.3)	54.1 (45.5 - 64.8)	53.9 (45.9 – 64.8)	0.99

*Kruskal Wallisov test (Post hoc test Conover), **χ^2 test

[1] Abbreviations: BMI - body mass index, WHR – waist-to-height ratio, WC - waist circumference, HC - hip circumference, MUAC - mid-upper arm circumference, FM - fat mass, PhA - phase angle, VF - visceral fat, FFM – fat-free mass, TBW - total body water, ECW - extracellular water, ICW - intracelular water, PMM - percentage of muscle mass

Supplementary Table S4. Correlations of anthropometric and body composition measurements with IPAQ-SF categories.

	**IPAQ1**[1]	**IPAQ2**[1]	**IPAQ3**[1]	**IPAQ4**[1]	**IPAQ5**[1]	**IPAQ6**[1]	**IPAQ7**[1]	**Total MET[1] score**
Height (cm)	0.099 (0.12)	0.052 (0.41)	-0.003 (0.97)	0.035 (0.59)	0.087 (0.17)	**0.172 (0.01)**	0.037 (0.57)	**0.157 (0.01)**
Weight (kg)	0.026 (0.68)	-0.005 (0.94)	-0.031 (0.63)	0.019 (0.76)	**-0.183 (<0.001)**	-0.019 (0.76)	0.040 (0.54)	-0.009 (0.89)
BMI[1] (kg/m^2)	-0.026 (0.69)	-0.039 (0.54)	-0.008 (0.90)	0.020 (0.76)	**-0.257 (<0.001)**	-0.103 (0.11)	0.003 (0.96)	-0.087 (0.17)
WHR[1]	-0.060 (0.35)	-0.056 (0.38)	0.033 (0.60)	0.009 (0.89)	-0.049 (0.44)	**-0.181 (<0.001)**	-0.043 (0.50)	-0.088 (0.17)

WC (cm)	-0.025 (0.69)	-0.023 (0.72)	-0.025 (0.70)	-0.005 (0.93)	-0.057 (0.37)	-0.076 (0.23)	-0.099 (0.12)	-0.076 (0.23)
HC (cm)	-0.024 (0.71)	-0.029 (0.65)	-0.058 (0.36)	-0.016 (0.80)	-0.024 (0.71)	0.040 (0.53)	-0.098 (0.12)	-0.044 (0.49)
MUAC (cm)	-0.025 (0.69)	-0.020 (0.75)	-0.024 (0.71)	0.022 (0.73)	0.012 (0.84)	0.023 (0.72)	-0.012 (0.85)	-0.003 (0.96)
FM[1] (%)	-0.108 (0.09)	-0.091 (0.15)	0.092 (0.15)	0.075 (0.24)	**-0.177 (0.01)**	**-0.158 (0.01)**	-0.021 (0.74)	**-0.140 (0.03)**
FM[1] (kg)	-0.056 (0.38)	-0.065 (0.31)	0.068 (0.29)	0.082 (0.20)	**-0.211 (<0.001)**	**-0.110 (0.09)**	-0.015 (0.82)	-0.084 (0.19)
VF[1] level	-0.081 (0.22)	-0.100 (0.13)	-0.104 (0.11)	-0.061 (0.35)	**-0.163 (0.01)**	-0.123 (0.06)	0.038 (0.56)	**-0.137 (0.04)**
FFM[1] (kg)	0.078 (0.22)	0.036 (0.58)	-0.061 (0.34)	-0.011 (0.86)	-0.072 (0.26)	0.071 (0.27)	0.037 (0.56)	0.066 (0.30)
TBW[1] (kg)	0.082 (0.20)	0.042 (0.52)	-0.067 (0.30)	-0.015 (0.81)	-0.075 (0.24)	0.075 (0.24)	0.033 (0.61)	0.066 (0.30)
ECW[1] (kg)	0.039 (0.55)	0.003 (0.96)	-0.081 (0.20)	-0.024 (0.71)	**-0.146 (0.02)**	0.010 (0.88)	0.075 (0.24)	0.004 (0.95)
ICW[1] (kg)	0.100 (0.12)	0.060 (0.35)	-0.113 (0.08)	-0.051 (0.42)	-0.080 (0.21)	0.082 (0.20)	0.082 (0.20)	0.064 (0.32)
PMM[1] (%)	0.076 (0.23)	0.034 (0.60)	-0.060 (0.35)	-0.011 (0.86)	-0.070 (0.27)	0.072 (0.26)	0.031 (0.63)	0.065 (0.31)
PhA[1] (^{0})	**0.186 (<0.001)**	**0.161 (0.01)**	-0.022 (0.74)	0.015 (0.81)	-0.039 (0.55)	0.077 (0.24)	0.017 (0.80)	0.113 (0.09)

* Data format: Rho (p-value)

[1] Abbreviations: IPAQ1 – number of days with vigorous activity, IPAQ2 – number of minutes in average spent in vigorous activity per day, IPAQ3 - number of days with moderate activity, IPAQ4 - number of minutes in average spent in moderate activity per day, IPAQ5 - number of days with continuous walking activity, IPAQ6 – number of minutes in average spent walking per day, IPAQ7 – time spent sitting per day (in hours), MET - metabolic equivalent of task, BMI - body mass index, WHR – waist-to-height ratio, WC - waist circumference, HC - hip circumference, MUAC - mid-upper arm circumference, FM - fat mass, VF - visceral fat, FFM – fat-free mass, TBW - total body water, ECW - extracellular water, ICW - intracelular water, PMM - percentage of muscle mass, PhA - phase angle.

Supplementary Table S5. Adherence to Mediterranean diet according to levels of physical activity.

**MDSS**	**Number (%) of participants**				*P**
	**Low level of PA (n=79)**	**Moderate level of PA (n=114)**	**High level of PA (n=59)**	**Total (n=252)**	
Grains	21 (27)	30 (26)	19 (32)	70 (28)	0.69
Potato	73 (92)	101 (89)	54 (92)	228 (90)	0.70
Olive oil	16 (20)	29 (25)	12 (20)	57 (23)	0.65
Nuts	20 (25)	32 (28)	17 (29)	69 (27)	0.88
Fresh fruit	23 (29)	26 (23)	10 (17)	59 (23)	0.26
Vegetables	10 (13)	27 (24)	8 (14)	45 (18)	0.11
Dairy	17 (22)	14 (12)	10 (17)	41 (16)	0.23
Legumes	32 (41)	61 (54)	27 (46)	120 (48)	0.20
Eggs	34 (43)	47 (41)	26 (44)	107 (42)	0.95
Fish	23 (29)	41 (36)	17 (29)	81 (32)	0.50
White meat	28 (35)	41 (36)	17 (29)	86 (34)	0.62

Red meat	34 (43)	42 (37)	22 (37)	98 (39)	0.66
Sweets	56 (71)	75 (66)	38 (64)	169 (67)	0.67
Wine (1 glass for women, 1-2 glasses for males)	18 (23)	21 (18)	8 (14)	47 (19)	0.39
MDSS total ≥14	6 (8)	9 (8)	3 (5)	18 (7)	0.78
MDSS total < 14	73 (92)	105 (92)	56 (95)	234 (93)	
MDSS total, median (IQR)	7 (5 – 9)	7 (5 – 10)	6 (4 – 9)	7 (5 – 10)	0.62**

*χ^2 test, **Kruskal Wallisov test

MDSS – Mediterranean Diet Serving Score

Supplementary Table S6. Correlations of MDSS components and total Mediterranean diet adherence with IPAQ-SF categories.

	**IPAQ1**[1]	**IPAQ2**[1]	**IPAQ3**[1]	**IPAQ4**[1]	**IPAQ5**[1]	**IPAQ6**[1]	**IPAQ7**[1]	**Total MET**[1] **score**
Total MDSS score	0.026 (0.68)	-0.029 (0.65)	-0.034 (0.59)	**-0.153 (0.02)**	0.022 (0.73)	-0.037 (0.56)	0.028 (0.67)	-0.092 (0.15)
Grains	0.105 (0.10)	0.040 (0.53)	0.004 (0.95)	-0.011 (0.86)	-0.026 (0.68)	-0.065 (0.30)	-0.041 (0.52)	0.041 (0.51)
Potato	0.002 (0.98)	0.008 (0.90)	-0.095 (0.13)	0.065 (0.30)	0.016 (0.80)	0.019 (0.77)	0.009 (0.89)	0.045 (0.48)
Olive oil	0.020 (0.75)	-0.037 (0.56)	-0.026 (0.68)	-0.104 (0.10)	-0.035 (0.58)	**-0.167 (0.01)**	0.114 (0.07)	-0.076 (0.23)
Nuts	0.060 (0.34)	0.045 (0.48)	-0.014 (0.82)	-0.051 (0.42)	0.112 (0.07)	-0.013 (0.84)	-0.029 (0.65)	0.026 (0.68)
Fresh fruit	-0.038 (0.55)	-0.091 (0.15)	-0.031 (0.62)	-0.112 (0.08)	-0.076 (0.23)	-0.122 (0.05)	0.054 (0.39)	**-0.136 (0.03)**
Vegetables	-0.026 (0.69)	**-0.135 (0.03)**	0.054 (0.39)	**-0.146 (0.02)**	0.122 (0.05)	-0.021 (0.73)	0.031 (0.63)	-0.116 (0.07)
Dairy	-0.040 (0.53)	-0.047 (0.46)	-0.062 (0.33)	-0.042 (0.51)	0.002 (0.97)	0.027 (0.67)	0.019 (0.76)	-0.069 (0.27)
Legumes	0.054 (0.39)	0.064 (0.31)	0.048 (0.45)	0.011 (0.87)	0.104 (0.10)	0.100 (0.11)	-0.037 (0.56)	0.095 (0.13)
Eggs	0.037 (0.56)	**0.152 (0.02)**	-0.022 (0.73)	-0.050 (0.43)	0.026 (0.68)	0.001 (0.99)	0.009 (0.89)	0.059 (0.35)
Fish	0.001 (0.99)	0.068 (0.28)	0.015 (0.82)	-0.010 (0.87)	0.015 (0.81)	0.038 (0.55)	**-0.159 (0.01)**	0.009 (0.88)
White meat	0.034 (0.59)	0.062 (0.33)	-0.066 (0.30)	-0.049 (0.44)	0.044 (0.48)	-0.065 (0.30)	0.010 (0.87)	-0.016 (0.80)
Red meat	-0.054 (0.39)	-0.032 (0.62)	0.032 (0.62)	-0.058 (0.36)	-0.089 (0.16)	-0.018 (0.78)	0.018 (0.77)	-0.054 (0.40)
Sweets	-0.032 (0.61)	-0.031 (0.62)	-0.013 (0.84)	-0.010 (0.88)	0.008 (0.90)	0.034 (0.59)	-0.092 (0.15)	-0.010 (0.87)
Wine (1 glass for women, 1-2 glasses for males)	-0.069 (0.28)	0.025 (0.69)	-0.094 (0.14)	0.043 (0.49)	0.006 (0.92)	-0.014 (0.83)	-0.052 (0.41)	-0.027 (0.67)

* Data format: Rho (p-value)

[1] Abbreviations: IPAQ1 – number of days with vigorous activity, IPAQ2 – number of minutes in average spent in vigorous activity per day, IPAQ3 - number of days with moderate activity, IPAQ4 - number of minutes in average spent in moderate activity per day, IPAQ5 - number of days with continuous walking activity, IPAQ6 – number of minutes in average spent walking per day, IPAQ7 – time spent sitting per day (in hours), MET – metabolic equivalent of task, MDSS – Mediterranean Diet Serving Score
